# Comparison of Athletes’ Proneness to Depressive Symptoms in Individual and Team Sports: Research on Psychological Mediators in Junior Elite Athletes

**DOI:** 10.3389/fpsyg.2016.00893

**Published:** 2016-06-17

**Authors:** Insa Nixdorf, Raphael Frank, Jürgen Beckmann

**Affiliations:** Department of Sport Psychology, Technical University of MunichMunich, Germany

**Keywords:** depression, junior elite athletes, team sports, individual sports, attribution

## Abstract

Depression among elite athletes is a topic of increasing interest and public awareness. Currently, empirical data on elite athletes’ depressive symptoms are rare. Recent results indicate sport-related mechanisms and effects on depression prevalence in elite athlete samples; specific factors associated with depression include overtraining, injury, and failure in competition. One such effect is that athletes competing in individual sports were found to be more prone to depressive symptoms than athletes competing in team sports. The present study examined this effect by testing three possible, psychological mediators based on theoretical and empirical assumptions: namely, cohesion in team or training groups; perception of perfectionistic expectations from others; and negative attribution after failure. In a cross-sectional study, 199 German junior elite athletes (*M*_age_ = 14.96; *SD* = 1.56) participated and completed questionnaires on perfectionism, cohesion, attribution after failure, and depressive symptoms. Mediation analysis using path analysis with bootstrapping was used for data analysis. As expected, athletes in individual sports showed higher scores in depression than athletes in team sports [*t*(197) = 2.05; *p* < 0.05; *d* = 0.30]. Furthermore, negative attribution after failure was associated with individual sports (*β* = 0.27; *p* < 0.001), as well as with the dependent variable depression (*β* = 0.26; *p* < 0.01). Mediation hypothesis was supported by a significant indirect effect (*β* = 0.07; *p* < 0.05). Negative attribution after failure mediated the relationship between individual sports and depression scores. Neither cohesion nor perfectionism met essential criteria to serve as mediators: cohesion was not elevated in either team or individual sports, and perfectionism was positively related to team sports. The results support the assumption of previous findings on sport-specific mechanisms (here the effect between individual and team sports) contributing to depressive symptoms among elite athletes. Additionally, attribution after failure seems to play an important role in this regard and could be considered in further research and practitioners in the field of sport psychology.

## Introduction

Although depression among elite athletes seems to be a topic of interest, empirical data on prevalence rates and research on mechanisms in this regard are still rare. However, recent results on depression prevalence in elite athlete samples are noteworthy and range between 4% (Schaal et al., 2011), 24% (Wolanin et al., 2016), 27% (Gulliver et al., 2015), and in some cases even up to 68% in the last 36 months (Hammond et al., 2013). Obviously, there is variability in prevalence estimates, which might be due to different assessment methods (questionnaire vs. interview), different assessment times (period of heavy exercise, recovery, or championship), or samples (different sport disciplines, gender etc.). In fact, recent reviews on this matter (Frank et al., 2013; Wolanin et al., 2015) suggest depression in elite athletes to be connected to sport-specific mechanisms and factors, such as injuries, overtraining, or exceeding stress. Consequently, such factors should be taken into consideration while assessing depression in athletes.

However, few such sport-specific factors are known. For example, Hammond et al. (2013) showed largely increased levels of depressive symptoms among swimmers during competition. Moreover, the study found performance failure to account for an increase in the levels of depressive symptoms. Besides the importance of failure, research showed injuries during the athletic career as predictors for depressive syndromes (Leddy et al., 1994). Leddy et al. (1994) found injured athletes to experience depression not only within 1 week after an athletic injury but also to have significantly higher depression scores even 2 months *post*-injury. Appaneal et al. (2009) found similar results with elevated depression scores from 1 week up to 1 month after injury when compared with healthy controls. There has been a number of evidence suggesting that sport-related concussions can lead to changes in emotional state (Hutchison et al., 2009) and might be connected to depression (Kerr et al., 2012). But while there might be a significant connection between concussions and depression, there is evidence suggesting that other sport injuries may have comparable or greater effects on mental health (Mainwaring et al., 2010). Besides the effect of acute injuries, the overtraining syndrome can also threaten the mental and physical health of an athlete and has been connected to depression in athletes (Puffer and McShane, 1992; Armstrong and Van Heest, 2002).

Recent findings also showed a sport-related effect, indicating that depressive symptoms vary by sport type. It has been repeatedly shown that athletes competing in individual sports were more prone to depressive symptoms than athletes competing in team sports (Schaal et al., 2011; Nixdorf et al., 2013). In a German sample, Nixdorf et al. (2013) found higher scores in depressive symptoms for athletes competing in individual sports than those competing in team sports. In a French sample, Schaal et al. (2011) found differences between sport disciplines indicating higher scores in esthetic sports (24%) and fine motor skill sports (18%) in comparison to team ball sports (8%). In North America, Wolanin et al. (2016) found that athletes competing in track and field had the highest rate of depression scores, while lacrosse players had significantly lower levels of depression. Although these authors do not explicitly address a differentiation into individual sports and team sports, their results further support the assumption that higher depression scores are found in disciplines with competitions based mainly on an individual performance.

From a psychological perspective, there are some reasonable arguments for athletes in individual sports to be at a higher risk for depression. In this regard, attribution of failure and success might be one such psychological difference. Hanrahan and Cerin (2009) showed that athletes in individual and team sports differ in style of attribution. In detail, athletes competing in individual-sport disciplines showed attribution with higher levels in the dimension “internality”. For positive events, individual-sport athletes showed attributions to be more internal, stable, and global. As the authors point out, it seems logical for individual-sport athletes to make more internal attributions as they do not have teammates which can be credited or blamed for results. For positive events, this style of attribution has potentially benefits in regard to performance or persistence (Hanrahan and Biddle, 2008). However, for negative events it can be a risk factor for depression and negative mood (Abramson et al., 1989). Internal attribution after negative events (failure) is associated with negative effect, such as guilt and shame (Tracy and Robins, 2004). Moreover, research on depression indicated that internal, stable, and global attribution after failure can lead to depression (e.g., Hull and Mendolia, 1991; Alloy et al., 2006). It is thus plausible that internal attribution can explain why athletes in individual sports might be at greater risk for depressive symptoms after failure.

Regarding cognition and attitudes, perfectionism is another plausible underlying mechanism. Perfectionism can be defined as a personal disposition characterized by striving for flawlessness and setting exceedingly high standards. Furthermore, it is accompanied by tendencies for overly critical evaluations of one’s behavior (see Frost et al., 1990; Hewitt and Flett, 1991b; Flett and Hewitt, 2002). The concept of a multidimensional personality disposition (Enns and Cox, 2002) has different aspects, which can be regarded as maladaptive and adaptive (Stoeber and Otto, 2006). Maladaptive aspects have been demonstrated to be linked to depression (Hewitt and Flett, 1991a). In athletes, research also discusses maladaptive and adaptive aspects (Gotwals et al., 2012). On the maladaptive side, perfectionistic concerns have been repeatedly linked to burnout in athletes (Hill et al., 2008; e.g., Hill, 2013; Madigan et al., 2015). One aspect of perfectionistic concerns is perfectionistic expectations from others, e.g., coaches, teammates, and parents (Enns and Cox, 2002; Stoeber et al., 2004). Such perfectionistic expectations appear as one possible aspect to showcase the difference between individual and team sports. Most athletes reaching for an elite level will probably perceive pressure to perform well and therefore experience perfectionistic expectation from outside. But whereas in team sports, responsibilities can more likely be diffused, identifiability can be greater in individual sports (Scanlan, 1984; Widmeyer et al., 1992). Data also show greater interest in athlete’s performance in individual sports from a motivational perspective (van de Pol et al., 2015). These circumstances are discussed in regard to research indicating higher levels of social anxiety (Norton et al., 2000) and trait anxiety in individual athlete compared to team-sport athletes (Martin and Hall, 1997). Following this argumentation, while individual- and team-sport athletes both experience perfectionistic expectations these might be more intensified for individual sports. However, differences between perfectionism and sport disciplines have not been examined, neither its possible mediating role toward depressive symptoms in contrast between team and individual-sport athletes.

Besides cognitive factors such as attribution or attitudes, social factors (cohesion or social support) are associated with depressive symptoms and its development (e.g., Alloy et al., 2006; Au et al., 2009). Therefore, low social support is connected with elevated depressive scores. The relevance of these social factors for depression in athletes has been demonstrated (Armstrong and Oomen-Early, 2009; Ohlert, 2012). Recent articles indicated that even in retired athletes low social support is connected to depression throughout and after the athlete’s career (Gouttebarge et al., 2015). In regard to cohesion in teams and training groups of individual sports, differences can be assumed. The presence of shared goals and interdependent structures, e.g., can strengthen cohesion in teams (Evans and Eys, 2015). Feedback from coaches, experience of failure during important competition, surroundings, and support might be different depending on the sport discipline. Therefore, higher cohesion in teams can be expected and potentially mediate differences in depressive symptoms across sport disciplines.

In summary, it can be stated that there are plausible arguments for negative attribution after failure, perfectionistic expectations from outside and cohesion to be important variables for the association between depressive symptoms, team sports, and individual sports. More specifically, these variables could potentially mediate the observed differences regarding depressive symptoms among individual- and team-sport athletes. For testing these assumptions, we examined these variables in a cross-sectional study among German junior elite athletes. It was assumed (i) that even in junior elite athletes we would find differences regarding depressive symptoms between athletes in individual and team sports. Therefore, we expect higher depressive scores among individual-sport athletes. Furthermore, we hypothesized (ii) that negative attribution after failure, perfectionistic expectations from outside, and cohesion would mediate the association between individual sports and depression. No specific assumption was made about which variable was most likely to mediate this effect. As not all connections between possible mediators and type of sport have been established by previous research, this has been tested according to the stepwise approach from Baron and Kenny (1986). However, mediation was tested using path modeling with bootstrapping to check for possible indirect effects and have more power and control over type I error rates (Hayes, 2009).

## Materials and Methods

### Participants

In a cross-sectional study, *N* = 199 German junior elite athletes (*M*_age_ = 14.96; *SD* = 1.56) participated and completed questionnaires on perfectionism, attribution, cohesion, and depressive symptoms. Originally, 295 junior elite athletes had participated, of which 199 completed all questionnaires and therefore were included in the present study. Participants were part of a scientific project which was reviewed, approved and financially supported by the German Federal Institute of Sport Science (*Bundesinstitut für Sportwissenschaft;* BISp) in order to investigate and help prevent depression and burnout in young elite athletes. Therefore, only junior athletes with high competition level (at least regional selection squad or members in professional junior development facilities) were included in the study. Participants came from different sport disciplines. Individual sports were: mountain bike (*n* = 16), badminton (*n* = 9), gymnastics (*n* = 5), swimming (*n* = 10), ice running (*n* = 19), and short track (*n* = 12). Team sports were: soccer (*n* = 113) and hockey (*n* = 15).

### Measures

#### Depression

Depressive symptoms in junior athletes were assessed with the widely used German version of the Center for Epidemiologic Studies Depression Scale (CES-D) from the National Institute of Mental Health (Radloff, 1977; Hautzinger et al., 2011). The CES-D is a short, self-report scale designed to measure depressive symptomatology in the general population. It was also repeatedly used to assess depressive symptoms among elite athletes (Yang et al., 2007; e.g., Armstrong and Oomen-Early, 2009; Junge and Feddermann-Demont, 2016). The 20 items are assessed on a scale ranging from 0 to 3. The scale is constructed, reliable, and standardized for the age range 11–90 years. The scale has been found to have high internal consistency (α = 0.89), which was in the present study α = 0.85.

#### Cohesion

Cohesion in team and individual athletes was measured using the German version of the Group Environment Questionnaire (GEQ; Carron et al., 1985) by Ohlert (2012). The GEQ is a widely used questionnaire to assess cohesion by four factors, namely group integration (related to task), group integration (social), individual attraction to group (related to task), and individual attraction to group (social). The widely used GEQ was translated, adapted, and validated by (*N* = 418) German athletes (Ohlert, 2012). Adaption of the German version allowed assessment of cohesion in team and individual sports. Therefore, 18 items with a nine-point Likert scale (strongly agree to strongly disagree) were used. The scale was found to be internal consistent with Cronbach’s alpha ranging between α = 0.74 and α = 0.78 for the four subscales. Overall, reliability was good in the present study with an internal consistency of α = 0.81.

#### Perfectionism

Perception of perfectionistic expectations from outside was assessed using the subscale of the German Version of the Multidimensional Inventory of Perfectionism in Sport (MIPS; Stoeber et al., 2004). The MIPS was developed following existing questionnaires dominating research in the field of perfectionism (e.g., Frost Multidimensional Perfectionism Scale; FMPS; Frost et al., 1990; Multidimensional Perfectionism Scale; MPS; Hewitt and Flett, 1991b). The scale consists of nine subscales which can be regarded as either adaptive or maladaptive (Stoeber et al., 2004). The subscale used in the present study has eight items on a six-point Likert scale covering experiences of perfectionistic expectations from outside (from the coach) and is regarded as rather maladaptive (see Ashby and Rice, 2002; Enns and Cox, 2002). The scale was validated and tested regarding its reliability in two studies indicating good internal consistency (study 1: Cronbach’s α = 0.94; study 2: Cronbach’s α = 0.86). Reliability was good in the present study with Cronbach’s α = 0.88.

#### Attribution after Failure

Attribution after failure was assessed using the relevant dimensions, such as internality, stability, and globality after the last failure according to the Sport Attributional Style Scale (SASS; Hanrahan and Grove, 1990). Athletes had to rate their personal cause for failure and success on the following dimensions: internality, stability, globality, personal controllability, external controllability, and intentionality on separate seven-point bipolar scales. The SASS was shown to have adequate psychometric properties (Hanrahan and Grove, 1990). For analysis in the present study, the sum score for the three dimensions, such as internality, stability, and globality for the last failure was used.

### Procedure

After review and approval of the BISp, written informed consent by athletes and parents of each participating athlete was provided. Data were assessed anonymously and pre-season in all sport disciplines with an online questionnaire battery. In case of interest or for further information on personal data, participants could use an individual code to access their individual data.

## Results

For replicating previous findings, athletes competing in team sports were compared with athletes in individual sports regarding depressive symptoms. A one-sided *t*-test revealed significant differences between the groups [*t*(197) = 2.05; *p* < 0.05; *d* = 0.30] with higher levels of depressive symptoms in athletes in individual sports (*M* = 11.55; *SD* = 7.67) than in team sports (*M* = 9.47; *SD* = 6.80).

Second, the hypothesized mediating variables negative attribution after failure, perfectionistic expectations from outside and cohesion were tested and results are shown in **Table 1**. Higher scores in individual sports are shown for the factor negative attribution after failure [*t*(197) = 3.87; *p <* 0.001]. Cohesion did not differ between team and individual sports and perfectionism differed contradictory to the hypothesis [*t*(197) = –3.57; *p* < 0.001].

**Table 1:** Comparison of athletes in team and individual sports regarding possible mediators.

	Individual sports	Team sports	*t*-Test
Perfectionism	*M* = 13.79; *SD* = 8.33	*M* = 18.01; *SD* = 8.05	*t*(197) = –3.57; *p* < 0.001
Cohesion	*M* = 94.79; *SD* = 23.16	*M* = 97.75; *SD* = 17.07	*^∗^t*(197) = –0.94; *p* = 0.174
Negative attribution after failure	*M* = 12.55; *SD* = 3.03	*M* = 10.77; *SD* = 3.14	*t*(197) = 3.87; *p* < 0.001

*t-Tests were one sided as specific direction of difference was assumed. As perfectionism was distributed contradictory to hypothesis, two-tailored t-test was performed post hoc. As variances regarding the variable cohesion were not equal, Welch T-Test (^∗^) was performed in this regard.*

Mediation analysis using path analysis with bootstrapping was used for data analysis. Therefore, the statistical program R using the package lavaan was employed. The categorical variable sport discipline (either team sports or individual sports) was included in the regression model as dummy coded variable (team = 0; individual = 1). As individual sports were coded with a higher value than team sports, the variable is called individual sports for easier interpretation of negative and positive pathways. For comparison between mediators, all scales were standardized and standardized path coefficients are reported. In addition, to illustrate associations between mediators and the dependent variable correlations between these variables were computed. In this regard, **Table 2** shows correlations between cohesion and depressive symptoms (*r* = –0.41; *p* < 0.001) as well as with negative attribution and depressive symptoms (*r* = 0.28; *p* < 0.001). Correlation between perfectionism and depressive symptoms was small (*r* = 0.14; *p* = 0.045). Also, inter-correlation between possible mediators was small (cohesion and negative attribution after failure; *r* = 0.19; *p* = 0.008) or absent (perfectionism and cohesion; perfectionism and negative attribution after failure).

**Table 2:** Pearson correlation between possible mediators and depressive symptoms.

	Cohesion	Negative attribution after failure	Perfectionism
Depressive symptoms	*r* = -0.41; *p* < 0.001	*r* = 0.28; *p* < 0.001	*r* = 0.14; *p* = 0.045
Cohesion	–	*r* = -0.19; *p* = 0.008	*r* = 0.01; *p* = 0.906
Negative attribution after failure	–	–	*r* = 0.00; *p* = 0.958

**FIGURE 1:**
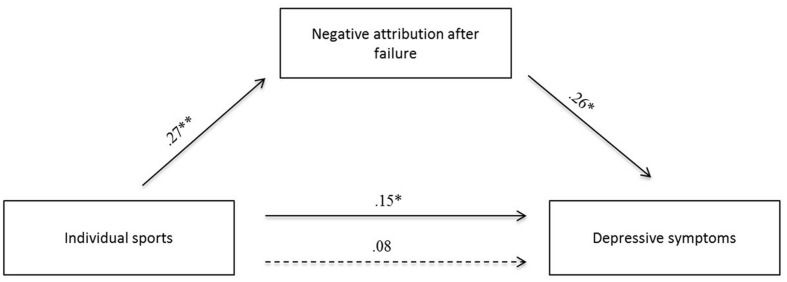
**Model of mediation path analysis with negative attribution after failure as mediator for the effect between sports and depressive symptoms (^∗^*p* < 0.05; ^∗∗^*p* < 0.001)**.

Regarding the mediation analysis, the overall model for negative attribution after failure as mediator between individual and team sports and depression is shown in **Figure 1**. Negative attribution after failure was associated with individual sports (*β* = 0.27; *p* < 0.001), as well as with the dependent variable depression (*β* = 0.26; *p* < 0.01). Mediation hypothesis was supported by a significant indirect effect (*β* = 0.07; *p* < 0.05) which showed a possible range between CI_0.95_ = 0.13, 0.02 with a 95% confidence interval (*R*^2^_med_ = 0.07). Therefore negative attribution after failure mediated the relationship between individual sports and depression scores.

Regarding the other possible mediators cohesion was significantly related to depression (*β* = –0.40; *p* < 0.001), as was perfectionism (*β* = 0.19; *p* < 0.05). However, as **Table 1** illustrates cohesion was not related to team sports and perfectionism was positively related to team sports, which was contradictory to the hypothesized mechanism in both regard. Thus, these variables did not meet essential criteria to serve as mediators (Baron and Kenny, 1986). Consequently, no analysis for mediating effects was performed.

## Discussion

The present study replicates the previously found difference in depressive symptoms between team- and individual-sport athletes (Schaal et al., 2011; Nixdorf et al., 2013; Wolanin et al., 2016), and results support the assumptions of previous findings on sport-specific mechanisms contributing to depressive symptoms among elite athletes. Athletes in individual sports showed higher scores in depressive symptoms than athletes in team sports, both in a non-clinical range in average. Along with other sport-specific mechanisms such as performance failure (Hammond et al., 2013) or injuries (Hutchison et al., 2009; Junge and Feddermann-Demont, 2016), the effects of individual vs. team sports should be taken into account when assessing clinical relevant prevalence in athletes to further explore the present sport-specific factors.

Whereas the previous studies referred to adult athletes, the present study was conducted with a relatively young athlete sample. Development of depression in the German general population is mostly evolving around adolescence and early adulthood (Jacobi et al., 2004). Therefore, the presence of this however small effect seems noteworthy and an increase in older samples seems likely. Furthermore, it indicates that possible underlying mechanisms for this effect are also sport inherent from an early stage.

Attribution after failure appears to be one such sport inherent factor that accounted for mediation in the present study. Thus, attribution seems to play an important role in explaining the different vulnerability to depression in team and individual sports. Since success and failure in individual sports are mostly based on the single athletes’ performance an internal attributional style is more common in individual sports than in team sports (Hanrahan and Cerin, 2009). Future research should develop this assumption by going into greater detail regarding the level of interaction in different sports (assuming a continuum from single performance, through added, to coactive and finally interactive performance). Attribution after failure can be compared across sport disciplines such as swimming (single performance), relays (added performance), rowing (coactive performance), and volleyball (interactive performance).

Besides depressive symptoms, other outcomes such as motivational or emotional aspects could be affected by an internal attributional style in athletes. Following the framework of Tracy and Robins (2004) on self-conscious emotions, the internal attribution could lead to stronger experiences of emotions such as pride (positive event) and guilt or shame (negative events) in athletes in individual sports. Therefore, further investigations on possible outcomes contrasting individual- and team-sport athletes could support existing theories on attributional style and deliver useful information for practitioners in the field.

Comparable to research on athlete burnout (Hill et al., 2008; Madigan et al., 2015), the present study found a connection between one maladaptive aspect of perfectionism (perfectionistic expectations from outside) with depression. Applying knowledge from the research on perfectionism and burnout to depression could be useful in this regard. Perfectionism had a positive relationship with team sports, with athletes in team sports being more prone to perfectionism than athletes in individual sports. Thus, the assumed relationship that individual athletes would experience higher levels of perfectionistic expectations due to their more obvious performance was clearly not supported. Recent research on burnout showed perfectionistic strivings to be connected to autonomous motivation and therefore prevent burnout (Jowett et al., 2013). Only perfectionism associated with controlled motivation should increase vulnerability to depression. However, Jowett et al. (2013) findings show once more that there is no unidimensional relationship between perfectionism and negative outcomes such as burnout or depression. Although in the present study a rather maladaptive aspect of perfectionism was used (Stoeber et al., 2004) this may also indicate that the construct as well as the assessment of perfectionism may be in need of further elaboration to clearly cover the different aspects associated with perfectionism.

Cohesion was associated with lower levels of depression in the present sample, leading to the assumption of cohesion being a protective factor for athletes. In line with previous findings (Armstrong and Oomen-Early, 2009; Gouttebarge et al., 2015), social factors might be important regarding depressive symptoms in athletes. However, no difference between individual- and team-sport athletes for cohesion was observed. This could be due to our sample. Also, it seems plausible that cohesion may not be the suitable factor to assess social connectedness and group dynamics. Evans et al. (2012) promote the investigation of group dynamics and social influence in individual sport by proposing a typology that distinguishes types of sport group environments according to levels of structural interdependence. Here, the individual athlete may still be exposed to similar cohesion effects as the team of athletes. Therefore, other variables such as coaching behavior and training environments could be important for the association between cohesion and depression.

As above mentioned, the present study assessed depressive symptoms in a relatively young sample. Goal of the study was to gather hints for underlying mechanisms with sport related connection in order to support prevention in this regard. Although differences in depressive symptoms were observed, results showed comparable means to the general population and most athletes were in a non-clinical range. Therefore, assumptions on the clinical relevance of this effect have to be further explored, using valid cut-off scores or clinical diagnosis by structured diagnostic interviews.

The present study is correlational in nature. Thus, causal implications cannot be drawn from the design. It is highly likely for the type of sport to be stable and can therefore be regarded as an early factor in a possible underlying sequence. However, no sequential order in regards to attribution or depression can be made. Thus results on mediator and outcome have no causal implication. Future research could investigate such causal mechanisms in prospective study designs. Nevertheless, practitioners could use these findings by considering attributional style and attribution after highly relevant events, especially in individual-sport disciplines and even in junior elite athletes in order to prevent negative reactions to failure such as depression.

## Author Contributions

IN, RF, and JB are a research group at the Chair of Sport psychology at the Technical University of Munich, Germany. The original research is part of the Ph.D.-Theses of IN and RF of which JB is the Ph.D. supervisor. Therefore, the conception and design of the work was a process done by all three authors in equal parts. The acquisition and analysis has been mainly lead by IN and RF. The interpretation of the data and the actual writing of the manuscript have been done by all three authors in equal parts. It is critically revised and approved to be published by IN, RF, and JB. All three authors agree to be accountable for all aspects of the work and ensure that questions related to the accuracy or integrity of any part of the work are appropriately investigated and resolved.

## Conflict of Interest Statement

The authors declare that the research was conducted in the absence of any commercial or financial relationships that could be construed as a potential conflict of interest. The reviewer KA and handling Editor declared their shared affiliation, and the handling Editor states that the process nevertheless met the standards of a fair and objective review.
